# Exercise Training as a Non-Pharmacological Therapy for Patients with Pulmonary Arterial Hypertension: Home-Based Rehabilitation Program and Training Recommendations

**DOI:** 10.3390/jcm11236932

**Published:** 2022-11-24

**Authors:** Mariusz Wojciuk, Zofia Dzięcioł-Anikiej, Katarzyna Kaniewska, Mariusz Ciołkiewicz, Diana Moskal-Jasińska, Anna Kuryliszyn-Moskal

**Affiliations:** 1 Department of Rehabilitation, Medical University of Bialystok, 24A M. Skłodowskiej-Curie St., 15-276 Bialystok, Poland; 2 Department of Clinical Phonoaudiology and Speech Therapy, Medical University of Bialystok, 37 Szpitalna St., 15-295 Bialystok, Poland

**Keywords:** PAH, pulmonary arterial hypertension, exercise training, home-based rehabilitation program

## Abstract

Pulmonary arterial hypertension (PAH) is a chronic and progressive disorder with a poor prognosis associated with non-specific symptoms, including general weakness, shortness of breath on exertion, and decreased muscle strength and endurance. Despite recent significant progress in the field of PAH therapy, many patients are still characterized by a dynamic course of the disease, a significant reduction in physical performance, a constantly deteriorating quality of life, and limited activity in everyday life. Thus, the main goal of PAH therapy is to ensure an acceptable level of quality of life as early as possible in the course of the disease, reduce the progression of symptoms and, if possible, improve the prognosis, which is still poor. The perception of the importance of activity and exercise has changed significantly in recent years, and rehabilitation dedicated to PAH patients is now considered to be one of the new adjuvant treatment options. Currently, there is insufficient data on what form, frequency, and intensity of exercise are required for the best results. Nevertheless, exercise training (ET) is necessary in order to reverse the accompanying PAH impairment of exercise capacity and, without additional clinical risk, to maximize the benefits of pharmacotherapy. This review summarizes the current state of knowledge on the rehabilitation of PAH patients and presents the available rehabilitation models. In addition, it includes a ready-to-use, illustrated, safe home rehabilitation program with recommendations for its use. Utilizing ET as an adjuvant treatment option to improve the functional capacity and quality of life of patients may enhance the clinical effectiveness of therapeutic management and contribute to the improvement of the quality of care for patients suffering from PAH. The beneficial effect of exercise training on the development of symptoms improves the clinical course of the disease, and a lower incidence of adverse events can lead to a reduction in health care expenditure.

## 1. Introduction

Pulmonary arterial hypertension (PAH) is an incurable [1,2] pathophysiological [1] condition associated with non-specific symptoms, including general weakness, shortness of breath on exertion, decreased muscle strength and endurance, leg fatigue, and peripheral edema, significantly affecting functional performance. In addition, patients may experience chest pain, coughing, palpitations, dizziness, and syncope, leading to reduced activity in daily living, significantly reduced physical performance, and a deterioration in the quality of life [3,4,5]. Reported complaints initially occur during exercise and later, along with the progression of the disease, at rest [5]. Thus, the main goal of PAH treatment is to ensure an acceptable level of quality of life as early as possible in the course of the disease [1], reduce symptom progression, improve functional class [6] and, if possible, improve prognosis [1,6,7,8], which remains poor [2,9]. This poses difficulties due to the non-specific symptoms significantly delaying the diagnosis of PAH [10]. Reported exercise intolerance may be initially ignored by patients due to reduced daily physical activity, poor commonly understood physical fitness, and as a result, a reduction of adequate cardiovascular and respiratory system efficiency [5]. A relationship between physical activity and the severity of PAH has been found [11]. Diagnosed patients severely reduce their physical activity, and more than half of them limit their normal activities of daily living [12]. Significantly, reduced daily physical activity in people with PAH has been shown to be associated with more severe symptoms reported by patients [13].

Numerous clinical studies have been carried out in order to enhance the knowledge and understanding of the pathophysiology of PAH, and yet this incurable condition, characterized by a high mortality rate, still requires a better transfer of new scientific knowledge to inform medical interventions [14,15]. Advanced specific therapies for pulmonary hypertension (PH), which resulted in clinical improvement and improved prognosis in PAH patients, still had little effect on physical performance. Exercise limitations in PH, caused by a variety of physiological mechanisms, have a profound effect on morbidity and mortality [16]. These limitations are the result of complex interactions between the circulatory and respiratory systems and the musculoskeletal system [17].

In recent years, the perceived importance of physical activity and exercise in patients with PAH has changed significantly [17,18], and exercise training (ET) dedicated to PAH (as adjuvant therapy in pharmacological treatment), which results in measurable improvement in patients’ health, is beginning to attract increasing interest among clinicians [19].

It is widely recognized that inequality in access to healthcare is a global concern [20]. Moreover, the availability of specialized rehabilitation programs for PAH patients and reimbursement of costs is largely limited to a small number of countries [15,19]. Therefore, the current research focuses on the development of an optimal rehabilitation model that is a compromise between the effectiveness, cost, and availability of therapy [18].

This paper summarizes the current state of knowledge on the rehabilitation of PAH patients and presents the available models of rehabilitation, which enables the selection of appropriate management strategies. In addition, it includes a ready-to-use, illustrated, safe home rehabilitation program (with effectiveness demonstrated in clinical studies) along with recommendations for its use.

## 2. Qualification for Rehabilitation

Pulmonary hypertension is a hemodynamic and pathophysiological condition associated with an increase in mean pulmonary arterial pressure (mPAP), assessed at rest by right heart catheterization (RHC). It may be caused by a primary increase in pressure in the pulmonary arterial system (pulmonary arterial hypertension) or a secondary due to an increase in pressure in the pulmonary venous and capillary systems (pulmonary venous hypertension). Pulmonary hypertension occurs mainly in the course of heart, lung, and pulmonary vessel diseases. In the face of the complex pathobiology of PH, thoughtful and responsible management of the therapy is essential, and thus the application of strict eligibility and exclusion criteria for rehabilitation. It should be emphasized that patients from all groups of PH in the World Health Organization Functional Class I–III (WHO-FC) receiving optimal medical therapy for the last three months can safely use ET. Due to the very small number of reports concerning the safety of ET in patients with an advanced course form of PH (WHO-FC IV), it is still recommended to undertake supervised physical activity only in rehabilitation centers dedicated to PH patients. Absolute and relative exclusion criteria are as follows: significant modification in the treatment of the main disease within three months, loss of consciousness within three months, severe uncontrolled lung and heart diseases, acute inflammation within four weeks, anemia, electrolyte and hormonal disorders within four weeks, other clinical situations that would make it impossible to participate in the rehabilitation program, and contraindications in accordance with the applicable standards of Comprehensive Cardiac Rehabilitation.

## 3. Main Components of the Exercise Training

Physical activity is one of the most important modifiable factors in our lifestyle and may significantly impact the prevention and treatment of cardiovascular diseases [21]. Exercise may modulate a variety of molecular mechanisms related to proliferation, apoptosis, oxidative stress, inflammation, thrombosis, proteolysis, and vasodilatation [19,22,23]. Without a doubt, ET is necessary in order to reverse the physical impairment that accompanies PAH and to maximize the benefits of pharmacotherapy [18] without apparent clinical risk [24]. Even a slight increase in normal activity levels can lead to beneficial effects on health, and individually adjusted ET can meet many potential therapeutic goals [24,25].

Unfortunately, despite the consensus in the relevant literature on the positive impact of the studied physical training programs and the recently made significant advances in understanding the pathobiology of PAH, the mechanisms underlying functional improvement in patients undergoing ET and its impact on long-term outcomes are currently not fully understood [19,22,24,25,26].

There is also insufficient data on what forms, frequencies, and intensities of exercise are required to obtain the most beneficial results [23,24,26,27,28]. The available literature shows that in addition to daily activity, regular endurance (or aerobic) training, strength training, and respiratory muscle training ought to be done [3,21,29,30,31]. Aerobic, resistance, and inspiratory muscle training, supported by individually adapted general conditioning training (also known as general fitness), stretching, body awareness training, neuromuscular relaxation techniques, positional relaxation, fascial therapy, etc., provide significant functional and physiological improvement [29,30].

### 3.1. Aerobic Training

The ways in which endurance training is carried out are generally consistent, despite differing in details [18]. Aerobic training is most often performed in the form of walking on a treadmill, walking outside or riding a bicycle ergometer (or both, which may increase the effectiveness of training) [18,19,30]. It is obvious that, despite the lack of studies that would test the effects of other popular exercise options, such as a recumbent stepper, cross trainer, upper body or horizontal ergometer, moderate to vigorous physical activity, regardless of the type of exercise, should positively affect physical performance [32]. Patients should also be encouraged to take walks outside of exercise sessions in order to minimize the daily reduction of physical activity [30].

Exercises can be carried out in a continuous or interval mode. In most of the rehabilitation programs developed for treating PAH, interval training models were used, which enables optimizing the efficiency of the training process [18].

Aerobic training should constitute a large part of the rehabilitation program. Currently, it is believed that aerobic exercise, performed 2–3 times a week, sufficiently improves patients’ 6-minute walk distance (6MWD) and provides a slight increase in peak VO_2_ value in cardiopulmonary exercise testing (CPET) [30]. However, it is likely that a higher frequency of aerobic exercise, performed 5 or more times a week, may increase physical fitness [28,30,33,34,35].

Initial sessions may consist of alternating same-duration exercises of moderate intensity followed by passive rest to avoid prolonged shortness of breath. As tolerance increases, the duration of intense exercise increases with it, eventually turning into a continuous phase of at least 30–45 min [30] with an intensity corresponding to approximately 60–80% of the HR (heart rate) achieved in an exercise test [25,28,35].

In addition to aerobic exercise, the inclusion of resistance exercise and inspiratory muscle training for 15 to 30 min as an element of a rehabilitation program has been associated with significant improvements in cardiopulmonary fitness and skeletal muscle profile [28,29,30,32].

### 3.2. Resistance Training

Resistance training may be an effective treatment option in patients suffering from PAH. Therefore, taking into account the way pulmonary hypertension affects skeletal muscle dysfunctions [3], some studies have utilized elements of resistance training as supplementary to endurance exercises [15,18,30]. In accordance with the recommendations of the American Heart Association, resistance training consisted mainly of low-level dumbbell training for large muscle groups [15,18]. Training loads should not exceed 50% of 1RM (one repetition maximum) for 10–15 repetitions per set. The Valsalva maneuver should also be avoided [25]. The available literature on PAH patients suggests that resistance exercises using body weight or with dumbbells weighing 500–1000 g for 15–30 min [29] sufficiently supplement aerobic training [25].

### 3.3. Breathing Training

Decreased strength and endurance of the inspiratory muscles are among the consequences (of high prognostic value) of the development of pulmonary arterial hypertension. This dysfunction results in increased dyspnea and fatigue during physical activity [18,30].

Breathing training may include breathing techniques, stretching exercises, strengthening the inspiratory muscles using resistance exercise devices, and activities such as yoga [29].

A common recommendation is that breathing training ought to consist of stretching, shaping and mobilizing the chest, re-educating the correct breathing patterns that enable ideomotor action, and strengthening the inspiratory muscles 5–7 days a week for up to 30 min [25,29]. Inspiratory muscle resistance training (as the main element of breathing training) may be performed with a small handheld device that blocks the patients’ airflow until a predetermined “threshold” pressure of 30–40% PImax (maximum inspiratory pressure) is created to effectively improve respiratory muscle strength and endurance [30,36,37].

### 3.4. Education and Psychological Support

Patient and family education is one of the essential elements of the rehabilitation program for PAH patients. The aim of these procedures is to learn about one’s own physical abilities [15], to be able to recognize one’s own limitations (beyond which the exertion may become excessive or dangerous) [25] and to learn how to cope with difficult situations [15]. Selected mental training techniques that improve physical and cognitive functions are also used [25]. Utilizing cognitive-behavioral therapy may facilitate the recognition of negative behavioral and/or thought patterns that lead to a deterioration in the quality of life in the realm of feelings and emotions and may be helpful in developing mechanisms of “coping” with these disorders. The aim of psychological training is also to broaden the knowledge, awareness, and acceptance of the disease and to facilitate intrapersonal, interpersonal, and professional adaptation to the current clinical state [25].

## 4. Setting of Rehabilitation Programs

Unfortunately, no clinically useful definition of exercise for pulmonary hypertension is yet available [4], while the variety of training forms and models increases the need for increasingly detailed analysis. Currently, research is focused on developing management strategies in order to both optimize the treatment effect and expand access to this kind of intervention [18,19]. These data are important for quantifying the various effects of ET and making it comparable to other general, supportive, or even targeted measures used in PAH [15].

Scientific evidence supports the notion that rehabilitation is a beneficial and safe non-pharmacological approach that should be considered in clinical strategies for the treatment of PAH [22,38], providing strong evidence for ET as an effective method of significantly improving the patient’s clinical condition [30]. However, the optimal model for rehabilitation in PH has not yet been established and may differ depending on the country or setting [18,26,39]. Despite this, there is a growing general interest among clinicians in more accessible methods of improving the functional status of patients has prompted them to increasingly use ET as adjuvant therapy.

According to experts, the best method of ET in patients with pulmonary arterial hypertension remains to be determined [27]. A number of options should be considered that take into account the strengths of the rehabilitation model, possible limitations, patients’ interest in improving their clinical condition, and whether funding for the rehabilitation process will be provided by private funds or public health care.

### 4.1. Inpatient Rehabilitation

The vast majority of evidence on inpatient rehabilitation originates from studies evaluating the protocol developed in Heidelberg (Germany). The implemented program included a 3-week hospital stay, followed by 12 weeks of home exercises remotely supervised by the PH expert facility [18,19].

The stationary exercise protocol was based on low-intensity interval aerobic training on a bicycle ergometer (10–25 min, 7 days a week) in a supervised and monitored environment. Additionally, 5 days a week, gait training was done with the assistance of specially trained physiotherapists (60 min). Its goal was to increase awareness of one’s physical limitations. Breathing training included learning methods to relieve shortness of breath (deep breathing exercises and postures that facilitate breathing), stretching and strengthening the breathing muscles, learning about different breathing techniques (such as pursed lip breathing), perception of one’s own body and breathing, and yoga (30 min). The training was supplemented with dumbbell (500–1000 g) resistance exercises of the main muscle groups (30 min). The intensive hospital protocol involved a minimum of 2 h of exercise per day [31,40]. After being discharged from the hospital, patients continued exercising as instructed in the individualized training brochure 5 days a week. Aerobic training on a cycle ergometer with an intensity close to the target heart rate, as well as breathing and resistance training for 15–30 min a day, were recommended. Patients were also required to undergo individual gait training twice a week (a total of ≥120 min of walking per week) [15,25,31].

However, inpatient rehabilitation is not widely available in many countries and potentially excludes patients whose lifestyle doesn’t permit them to follow this approach [15,19,39,41]. It is also difficult to replicate the intensity of this exercise program in routine clinical practice due to limited resources and personnel [42]. Reports from various research teams point out that following the inpatient rehabilitation approach in countries where it has not been implemented yet, would have a serious impact on healthcare costs [39]. It may be concluded that, mainly for this reason, service providers do not approve of the implementation of inpatient rehabilitation. However, it is highly likely that any changes in the approach to rehabilitation in pulmonary hypertension will require a costly and systemic extension of physiotherapeutic care as well as comprehensive education and increased access to specialists in this field [39].

### 4.2. Outpatient Rehabilitation

Due to the above-mentioned limitations, potential outpatient and home exercises for pulmonary hypertension [17,33,41,43,44,45] have been proposed as an alternative to rehabilitation in the inpatient model.

The studies conducted so far have been based mainly on 2–3 supervised sessions per week for about 10–12 weeks [15,18,27,41]. The programs consisted of different combinations of exercises, including interval and continuous endurance training (walking on a treadmill, cycle ergometer, walking outside, 10–60 min), resistance training (lower and upper limbs with light loads, 10–15 min), breathing exercises (10–30 min) and education, consisting of individualized counseling (psychological, dietary, occupational therapist) and group sessions with other patients (60 min) [18,27,32,43].

It should be noted that outpatient services are substantially cheaper [18]. The similarity of the basic elements of PAH rehabilitation to those provided as part of pulmonary or cardiac rehabilitation programs (endurance training, resistance training) suggests that people suffering from PAH could benefit from routine rehabilitation programs to improve the practical availability of these services [46] and maximize training efficiency while minimizing the impact of rehabilitation on the daily life of these patients [43]. This is due to one of the most serious problems in the rehabilitation of pulmonary arterial hypertension, which may prove insurmountable in most reference centers. In addition to funding, gathering a sufficiently large local group of patients (who are usually geographically dispersed) requires a high enough population density in order for PAH-specific outpatient rehabilitation to be implemented in practice. Therefore, records of outpatient rehabilitation of patients with pulmonary arterial hypertension were also based on more widely available, non-specific programs of pulmonary, cardiological, and even general rehabilitation [18].

### 4.3. Home-Based Rehabilitation

The literature review indicates that the outpatient rehabilitation model for PAH patients has a clear advantage over the inpatient model in terms of feasibility in a variety of health systems [44]. However, it has certain limitations, which, apart from geographic barriers and a lack of qualified personnel [15,17,39,44], include long waiting times for access to rehabilitation [39,44]. A home-based exercise training program eliminates the above-mentioned problems [44] and, as it has been demonstrated in the Cochrane review [47] on cardiac rehabilitation, it is comparable in effectiveness to a closely supervised inpatient program.

Currently, there is very little data available on the rehabilitation of PAH patients at home [18], even though this form of rehabilitation has been proven [17,44,48,49] to have a similarly high safety profile as rehabilitation in the inpatient model.

The available home rehabilitation protocols usually covered a 12-week rehabilitation program consisting, in its main part, of interval or continuous aerobic training (initially 5–10 min, ultimately 30–45 min, 5–6 days per week), resistance exercises (initially general fitness body weight exercises, and with an improvement of fitness levels, followed by a progressive increase in the number of repetitions, series, and loads, which ultimately oscillated between 500 and 1000 g, 2 days per week), breathing training (to mobilize the chest, improve the respiratory rhythm, emphasize diaphragmatic breathing, and strengthen the respiratory muscles), stretching, neuromuscular relaxation and body awareness techniques (5–10 min, 5–6 days per week). Training intensity ranged from 4 to 6 on the 10-point Borg scale or with the target intensity set at 65–75% of the heart rate reserve. The training was supplemented by educational and motivational elements utilized during in-person meetings with a physiotherapist or during weekly telephone calls [17,44,48,49].

The summary of the data on home rehabilitation program models is presented in Table 1.

It should be emphasized that the model of a home-based rehabilitation program enables a greater number of patients to benefit from this additional adjuvant therapy without excessively increasing the financial burden of the payer (as is the case in the inpatient rehabilitation model). The home-based rehabilitation program also makes physiotherapeutic intervention possible without costly adjustments to the facilities, infrastructure, and resources of a healthcare provider that is not a reference center for pulmonary hypertension. The developed training model ensures overcoming geographical barriers resulting from the wide dispersion of patients and minimizes costs (which significantly hinder the implementation of the rehabilitation program in the case of the outpatient model) borne by patients and their families. It also enables patients who, despite their illness, are still able to actively fulfill their local social roles (work, household duties, caring for the family, etc.) to participate in the rehabilitation process. The possibility of adjusting the time and place of training does not prevent the participants from undertaking planned daily activities related and increases the probability of implementing their training program into their daily schedule, exceeding the duration of the training protocol [48].

A detailed ready-to-use home-based physical training program (in the form of a brochure with photos along with a detailed description of exercises and training recommendations), the feasibility, effectiveness and safety of which has been verified in clinical trials [48] is attached to this study (Supplementary File S1).

## 5. Notes on Implementing the Home-Based Rehabilitation Program

The training begins with the initial phase (5–10 min) in order to prepare the cardiovascular and motor systems for increased exertion. The warm-up consists of 15 exercises covering all parts of the body. It takes the form of general fitness exercises of individual muscle groups using one’s own body weight (and with the improvement of muscle strength in resistance training with a target load within the range of 500–1000 g) and resistance training of inspiratory muscles using a simple Pulmogain, RespiTrain trainer (Moretti, Italy). Alternatively, a more advanced respiratory trainer can be used, e.g., Threshold IMT (Philips Respironics Inc, Pennsylvania, United States), with a load progression starting at 30–40% [36] to 65–70% [49] PImax. The main portion of the rehabilitation program comprises interval walking training (lasting at least 30 min), interrupted every 2 min with one of the five breathing exercises performed in the prescribed sequence. Interval aerobic training and breathing exercises reduce shortness of breath and fatigue at training, improve exercise tolerance, reduce breathing effort, correct breathing patterns, increase the mobility of the chest and diaphragm, strengthen the respiratory muscles, and increase lung volume. Exertion ends with a cooldown phase with relaxation and stretching exercises (5–15 min) that allow for a transition from training to a resting state by reducing heart rate, respiratory rate, and temperature. It prevents a sudden drop in blood pressure and prevents pre-syncope [48].

As safety needs to be ensured while exercising at home, it is recommended that the intensity of exercise should be rated at 4–6 on the 10-point Borg scale, and the training heart rate should be between 60–75% of heart rate reserve based on baseline parameters from CPET. Where available, it is recommended that patients use a pulse oximeter during training to stop or limit their exercise if they reach a heart rate of >120 beats per minute or a drop in SaO_2_ < 85%, as well as experience subjective symptoms of exercise intolerance (dizziness, pain, severe shortness of breath, fatigue, etc.) or perceive exertion at >6 on the Borg scale. In case of desaturation, the use of oxygen supplementation is recommended (considered to improve exercise capacity), which increases the intensity of exercise, and thus the effects achieved due to training [18,19,28,35]. Patients dependent on home oxygen therapy should exercise with portable oxygen concentrators.

The desired effects may be achieved by carrying out the training throughout the period of 12–24 weeks once a day, at least five times a week, which may result in the rehabilitation results lasting even 3–6 months after completing the exercise program [48,50]. The slightly modified weekly exercise program (Table 2) proposed by Butāne et al. [45] may prove useful in that regard.

The optimal target duration of training is 45–60 min, and the patient should reach this duration within the first 2 weeks, if possible. Training progression should be expressed in parameters that demonstrate the increasing number of repetitions and series of each exercise, resistance during skeletal and respiratory muscle training, training unit time, pace and distance in aerobic training. In order to ensure safety, it is advisable that family members or carers assist the patient during each training session.

Before the rehabilitation program begins (during an individual meeting of the patient and his family with a doctor or physiotherapist), participants should familiarize themselves with the training guidelines and the use of activity monitors or of the respiratory muscle trainer, individual contraindications to exercise, instructions and recommendations in the event of adverse events during or after training and learn to perform all the exercises correctly. Particular attention should be paid to the point of undertaking controlled physical activity and its benefits. The role of systematic cardiac rehabilitation should be emphasized. Patients treated with parenteral prostacyclins in the form of continuous infusions both subcutaneously and intravenously via external or implantable pumps, performing the rehabilitation program, should be additionally educated on the need for increased caution while exercising in order not to damage the device or system.

In addition, patients undergoing training should receive a self-control diary (Supplementary File S2) in order to collect data on each training day so that the implementation of the exercise program may be monitored. The parameters recorded both before and after training (heart rate, blood pressure, dyspnea, and fatigue on the 10-point Borg scale, exercise time, and the number of steps taken during training based on a reading from an accelerometer or smartphone application) allow for the long-term progression of the rehabilitation to be monitored and increase the motivation to exercise. It should be emphasized that patient education, high-quality training materials and user-friendly physical activity monitoring equipment can ensure optimal self-monitoring [45].

Due to the fact that the regularity of exercise plays an important role in the final result of the training program, participants should be under constant supervision by telephone (at least once a week) in order to verify compliance with the recommended rehabilitation protocol and overall mobilization to maintain the training regime. Regular monitoring of those doing the exercises ensures the optimization of effects and minimizes the risk of adverse events [26].

## 6. Conclusions

The beneficial effects of physical training include improvement in the development of symptoms and in the clinical course of the disease [15,27]. Consequently, a lower incidence of adverse events may lead to a reduction in healthcare costs in the long term. Moreover, the benefits associated with physical training, greater patient awareness, and gaining the ability to self-manage the disease may also measurably affect the financial burden of the payer [15,25]. Utilizing home-based physical exercise as an adjuvant treatment option to improve patients’ functional abilities and quality of life may enhance the clinical effects of therapeutic management and improve care quality for patients with PAH. This form of rehabilitation, which is currently the only realistic treatment option for PAH patients in most countries, significantly increases the availability of physiotherapeutic care for this group of patients.

## Figures and Tables

**Table 1:** The summary of home-based physical training program models.

Study		Training Program	
1.	**Brown** [44] (2018) PAH (*n* = 11) USA (Indianapolis)	12 weeks 6 days per week	- walk training (45 min); - 65–75% of the HR reserve.
2.	**Babu** [17] (2019) all PH (*n* = 34) India (Manipal)	12 weeks 3–5 days per week	Warm-up (5 min): - stretching exercises; - breathing exercises; - hand and foot exercises. Main part: - general fitness exercises and resistance training, initially 1–2 series of several repetitions as part of subjective fatigue assessed in accordance with the Borg scale at 4–6/10, with progression in the number of repetitions and the number of series, as well as loads up to 500–1000 g; - walk training (initially 5–10 min, ultimately > 30 min, 4–6/10 or 12–13/20 on the Borg scale). Final part: - relaxing exercises (5–10 min); - education (PulHMan manual); - the entire session lasted from 10–20 min to 30–40 min at the end of 12 weeks.
3.	**Wojciuk** [48] (2021) PAH (*n* = 16) Poland (Bialystok)	24 weeks 5–7 days per week	Warm-up (5–10 min): - general fitness exercises; - resistance training of inspiratory muscles using Pulmogain, RespiTrain breathing trainer devices. Main part: - interval walk training; interspersed with breathing exercises every 2 min (>30 min, 4–5/10 on the Borg scale, 60–70% of HR reserve). Final part: - relaxing and stretching exercises (5–15 min); - education and motivation; - duration of the entire session: 45–60 min.
4.	**Butāne** [49] (2022) PAH (*n* = 11) Latvia (Riga)	12 weeks 5 days per week	Monday: aerobic training, breathing exercises, neuromuscular relaxation. Tuesday: upper body resistance exercises, breathing exercises, neuromuscular relaxation. Wednesday: rest. Thursday: aerobic training, breathing exercises, neuromuscular relaxation. Friday: lower body resistance exercises, breathing exercises, neuromuscular relaxation. Saturday: aerobic training, breathing exercises, neuromuscular relaxation. Sunday: rest. - continuous aerobic training: e.g., walking, bicycle ergometer (20–40 min, 3 days per week), perceived exertion as 5–6/10 on the Borg scale and sustained SaO_2_ or a decrease of no more than 5% from baseline; - breathing training with PHILIPS Threshold IMT breathing trainer (repetitions from 3 × 3 to 3 × 7 in each set; resistance (from 30% to 65–70% from max) and relaxation techniques (5–10 min, 5 days per week); - resistance training (initially general fitness body weight exercises, followed by load progression up to 500–1000 g, 5–6 exercises, 5–10 repetitions each, 2 days per week); - education and motivation.

HR—heart rate; PAH—pulmonary arterial hypertension; PH—pulmonary hypertension; SaO_2_—blood oxygen saturation.

**Table 2:** Modified description of the exercise program.

Day of the Week	Components of the Exercise Training
Monday	Interval aerobic training Breathing training and relaxation
Tuesday	Resistance training Interval aerobic training Breathing training and relaxation
Wednesday	REST
Thursday	Interval aerobic training Breathing training and relaxation
Friday	Resistance training Interval aerobic training Breathing training and relaxation
Saturday	Interval aerobic training Breathing training and relaxation
Sunday	REST

Based on: Butāne, L. [45] (2019).

## Supplementary Materials

The following supporting information can be downloaded at: https://www.mdpi.com/article/10.3390/jcm11236932/s1, File S1: Home-based rehabilitation program—for patients with pulmonary arterial hypertension; File S2: Self-control diary.
