# Supplementary material for: Exercise Training as a Non-Pharmacological Therapy for Patients with Pulmonary Arterial Hypertension: Home-Based Rehabilitation Program and Training Recommendations
Source: J Clin Med. 2022 Nov 24;11(23):6932. doi: 10.3390/jcm11236932 (PMC9736155; doi:10.3390/jcm11236932)
Supplement: Supplementary file 1 [file jcm-11-06932-s001.zip › Supplementary File S1.pdf]

HOME-BASED REHABILITATION PROGRAM
- FOR PATIENTS WITH PULMONARY ARTERIAL HYPERTENSION

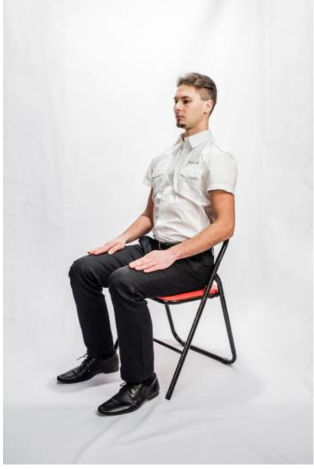

WARM-UP (5-10 MINUTES)

Sit down on a chair and assume the correct starting position for the exercises. Move close to the backrest, place your feet on the ground, hip-width apart. Straighten your back, pull your stomach in, but without holding your breath. Pinch your shoulder blades down and draw your chin back, but without lifting your face up. Try maintaining this position during subsequent exercises. Breathe freely, inhale through the nose and exhale in long slow exhalations through your mouth.

Do 8 – 10 – 12 repetitions of each exercise, depending on your abilities. During resistance training, use dumbbells, weights on ankles and wrists, or water bottles. Perform resistance exercises in 2 – 3 series of 10 – 15 repetitions. Try making every movement precisely and to its fullest extent. Do the exercises rhythmically but take your time!

1. Breathing exercise with the RespiTrain device. Follow the operating instructions for the device. Place the device at the level of your mouth. Breathe freely before exercising. Close your lips around the mouthpiece and inhale until the three balls inside the chambers rise. Keep the balls in the air for as long as possible. Remove the mouthpiece from your mouth and slowly breathe out through narrowed lips. Repeat the exercise 6 – 8 times. *This exercise can also be done with the Threshold IMT device.

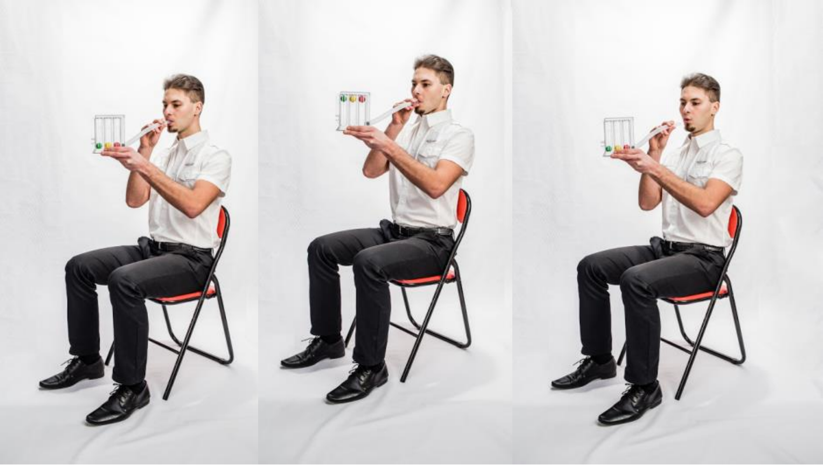

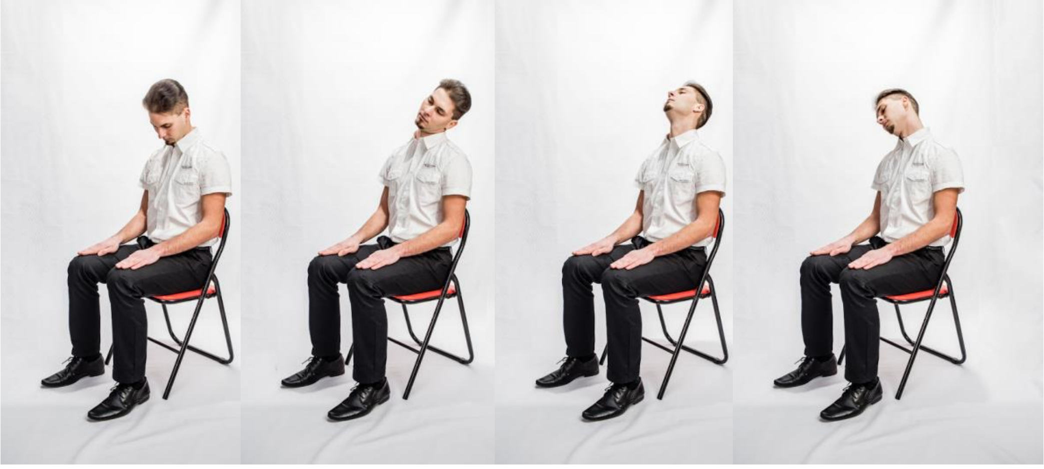

2. Head rotations. Make slow, ample head turns to the right and left. Keep the upright position and remember not to tilt your head too much to the back. Do the exercise 5-6 times to the left and 5-6 times to the right.

3. Forward and backward shoulder circles. Hands should be resting freely on your thighs. Make an ample backward circular movement of the shoulders by lifting them up, pinching the shoulder blades, lowering the shoulders, and extending them forward. Take care to make the movements as ample as possible. Maintain your upright position. Relax your muscles by shaking your arms along the torso. Do this exercise in the opposite direction.

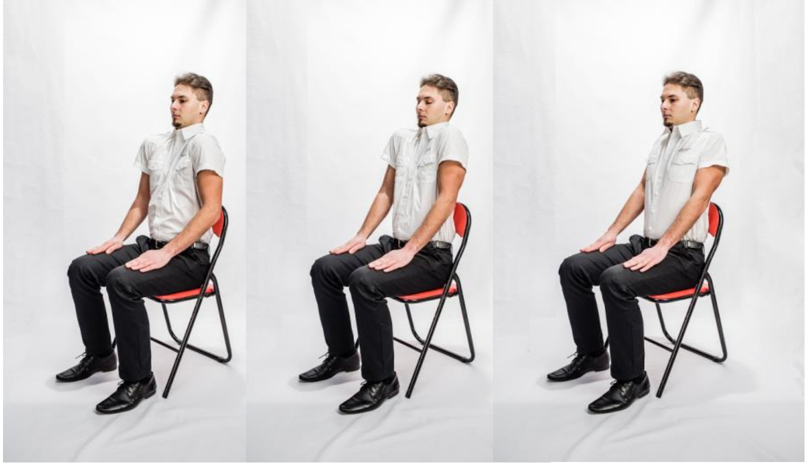

4. Bending and straightening the arms. Raise the right upper limb facing upwards while re-tracting the left limb, moving it close to the body. Perform this exercise alternately. *Perform this exercise with an additional load of 500 – 1000 g in resistance exercises!

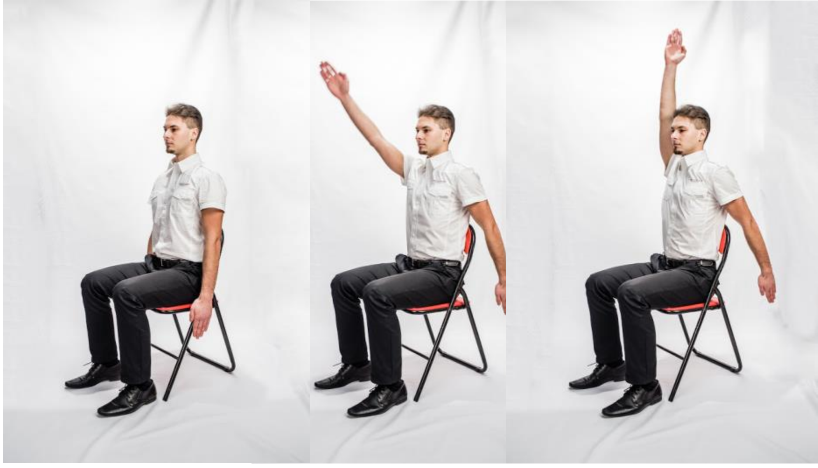

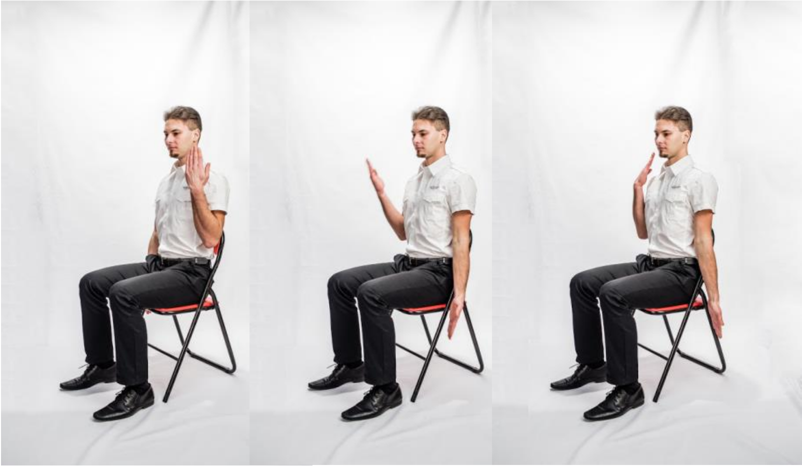

5. Flexing and straightening the forearms. The arms hang loose along the torso. Alternately bend your left and right arm at the elbow so that your palm touches the shoulder on the same side. *Perform this exercise with an additional load of 500 – 1000 g in resistance exercises!

6. Breathing exercise with the RespiTrain device. Follow the operating instructions for the device. Place the device at the level of your mouth. Breathe freely before exercising. Close your lips around the mouthpiece and inhale until the three balls inside the chambers rise. Keep the balls in the air for as long as possible. Remove the mouthpiece from your mouth and slowly breathe out through narrowed lips. Repeat the exercise 6-8 times. *This exercise can also be done with the Threshold IMT device.

7. Wrist circles. Elbows are bent and held close to the body. Make ample circling movements of the wrists in and out. *Do the exercise with extra weights held in your hands during resistance exercises!

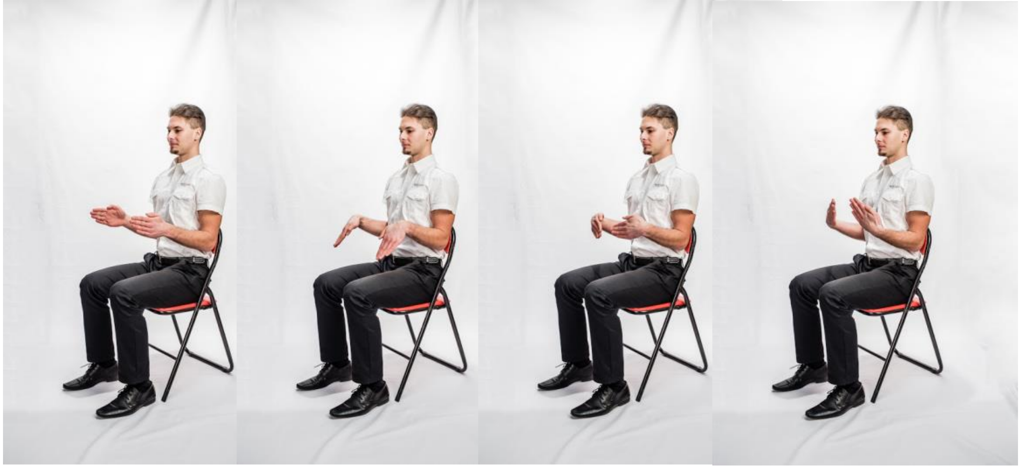

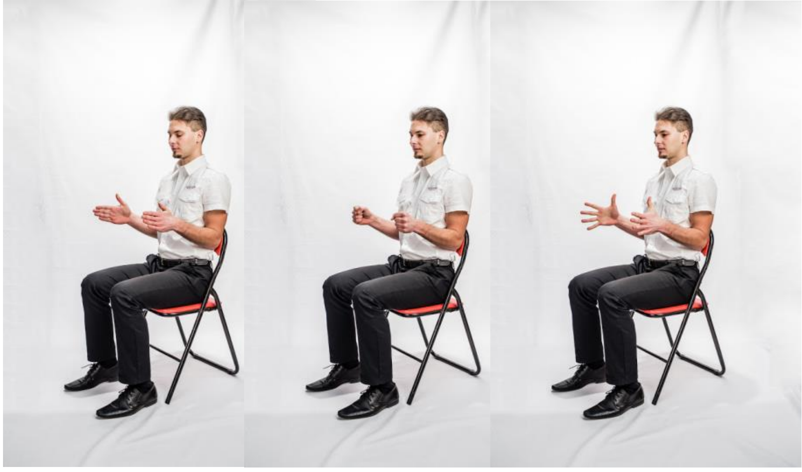

8. Bending and straightening the fingers. Do not change your position from the previous exercise. Continue sitting in the correct upright position with your elbows bent and held close to your body. Squeeze your fists vigorously and then straighten your fingers.

9. Seated marching. Grab the seat of the chair with your hands and stabilize your upright position. Alternately, lift your left and right knee high. Make sure that one foot is always in contact with the ground when doing this exercise. *Perform this exercise with an additional load of 500 – 1000 g in resistance exercises!

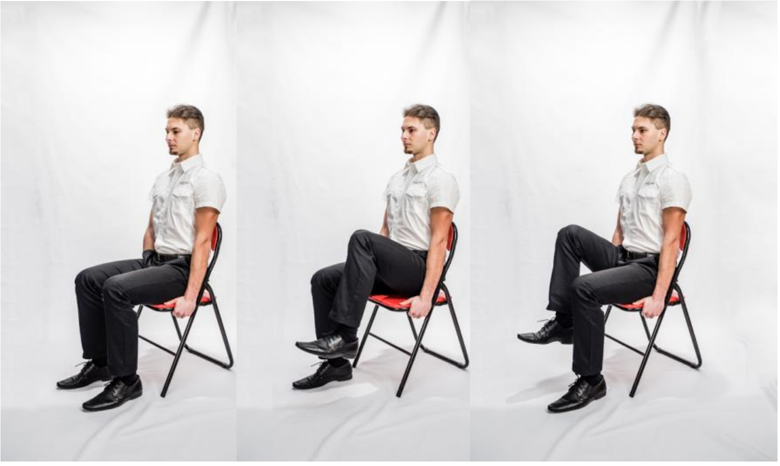

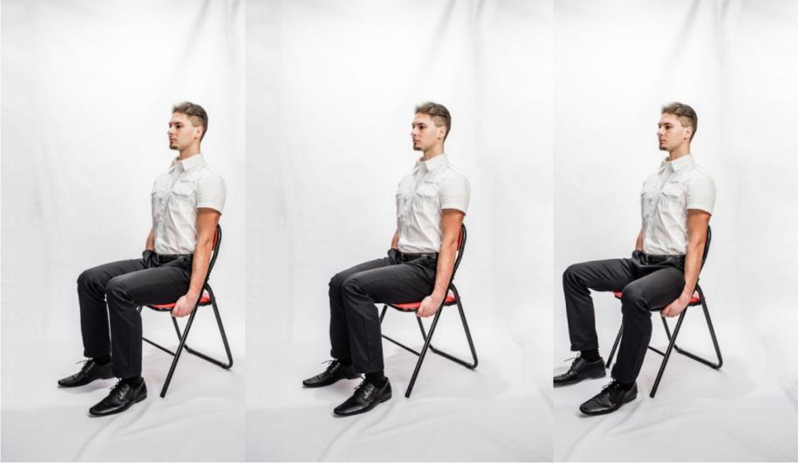

10. Joining knees. Position your feet hip-width apart. Bring your knees together, then spread them apart. Try not to move your feet.

11. Breathing exercise with the RespiTrain device. Follow the operating instructions for the device. Place the device at the level of your mouth. Breathe freely before exercising. Close your lips around the mouthpiece and inhale until the three balls inside the chambers rise. Keep the balls in the air for as long as possible. Remove the mouthpiece from your mouth and slowly breathe out through narrowed lips. Repeat the exercise 6 – 8 times. *This exercise can also be done with the Threshold IMT device.

12. Bending and straightening the knees. Grab the seat of the chair with your hands and stabilize yourself in an upright position. Extend your left leg forward and put it on your heel. Move the right leg backwards under the chair and rest it on your toes. Rhythmically alternate movements of the limbs to change their position. *Perform this exercise with an additional load of 500 – 1000 g in resistance exercises!

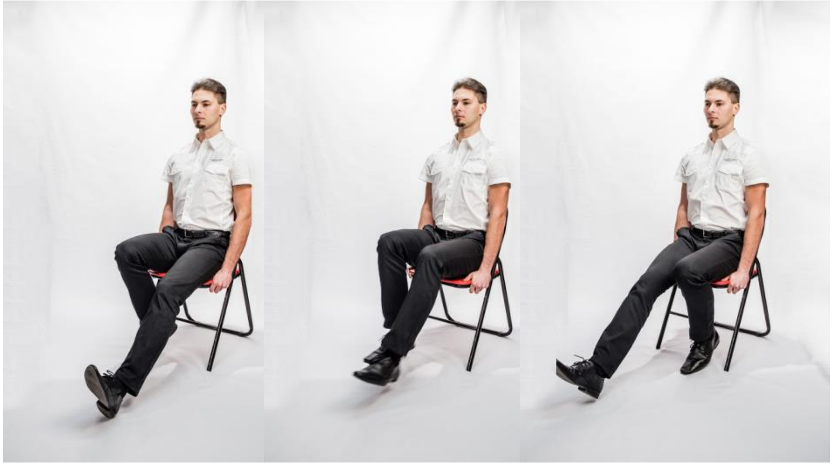

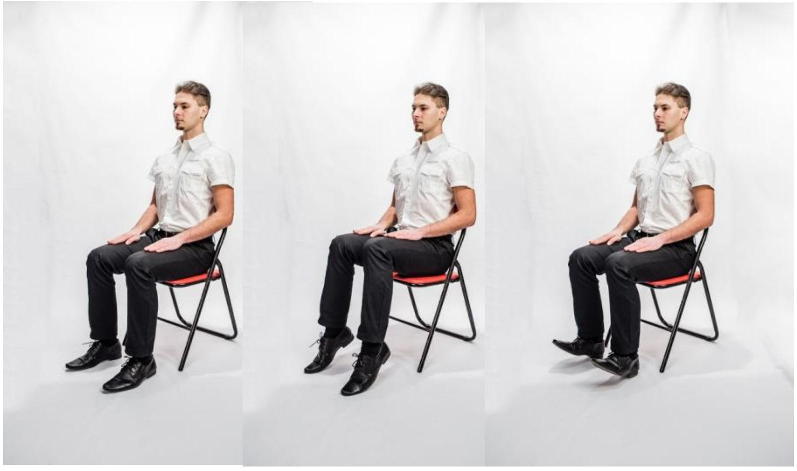

13. Bending and straightening the feet. Place your feet on the ground, hip-width apart. Alternately, perform toe and heel raises. *Perform this exercise with an additional load of 500 – 1000 g in resistance exercises!

14. Foot circles. Place your feet on your heels in front of you. Make ample circular movements in your ankles both in and out.

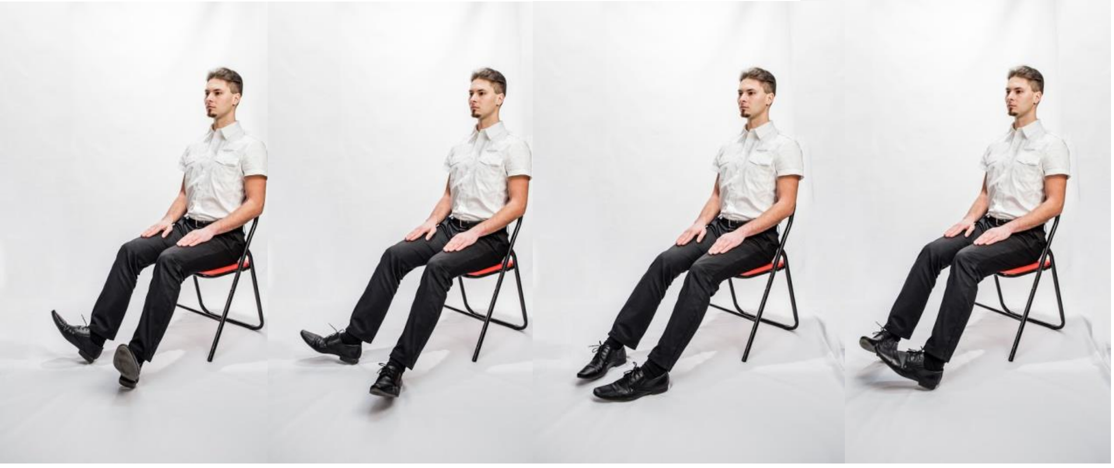

15. Breathing exercise with the RespiTrain device. Follow the operating instructions for the device. Place the device at the level of your mouth. Breathe freely before exercising. Close your lips around the mouthpiece and inhale until the three balls inside the chambers rise. Keep the balls in the air for as long as possible. Remove the mouthpiece from your mouth and slowly breathe out through narrowed lips. Repeat the exercise 6 – 8 times. *This exercise can also be done with the Threshold IMT device.

After this part of the training, your heart rate should increase by a few to a dozen beats per minute. Your breathing should deepen which means that you are ready to do aerobic exercise.

MAIN PART (AT LEAST 30 MINUTES)

Treadmill/outdoor march. Endurance training is an important element of your rehabilitation. Begin with a leisurely walk, and gradually increase your walking time to at least 30 minutes. Do this exercise no less than 5 times a week. The marching pace should make your breathing faster, but it shouldn't cause shortness of of breath. When walking, the accelerated breathing should allow for speaking while making singing impossible.

Your energetic, rhythmic march should be interrupted every 2 minutes with one breathing exercise to be done in sequence during the following breaks. Do the exercises while seated (following the instructions below), and when you are able to, while standing or walking slowly. Remember that we should always inhale through the nose, and exhale slowly through the narrowed mouth. Don't skip exercises!

ATTENTION! Aerobic training can also be done using a cycle ergometer, and a recumbent stepper, cross trainer, manual or horizontal ergometer, and Nordic walking may be used to add variety to the training.

Breathing exercise 1. Raise your shoulders while inhaling deeply through your nose. Lower them slowly as you exhale deeply through narrowed lips. Repeat the exercise 6-8 times.

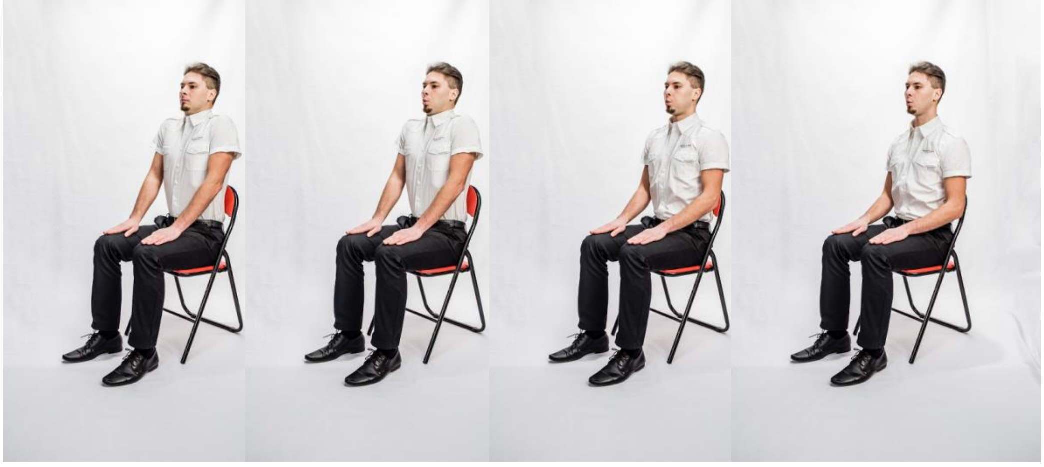

Breathing exercise 2. While inhaling deeply through your nose, raise your straight arms forward. Then, lower them sideways as you exhale deeply through narrowed lips. Repeat the exercise 6-8 times.

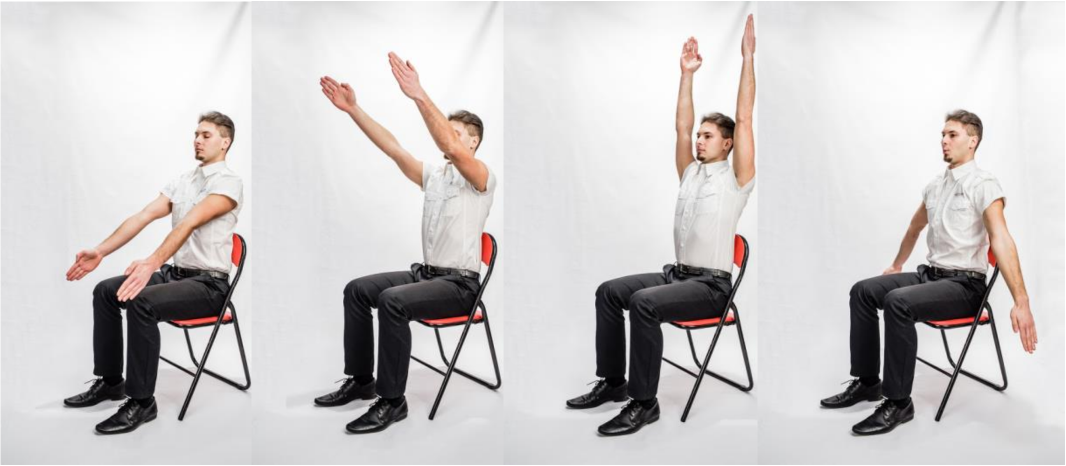

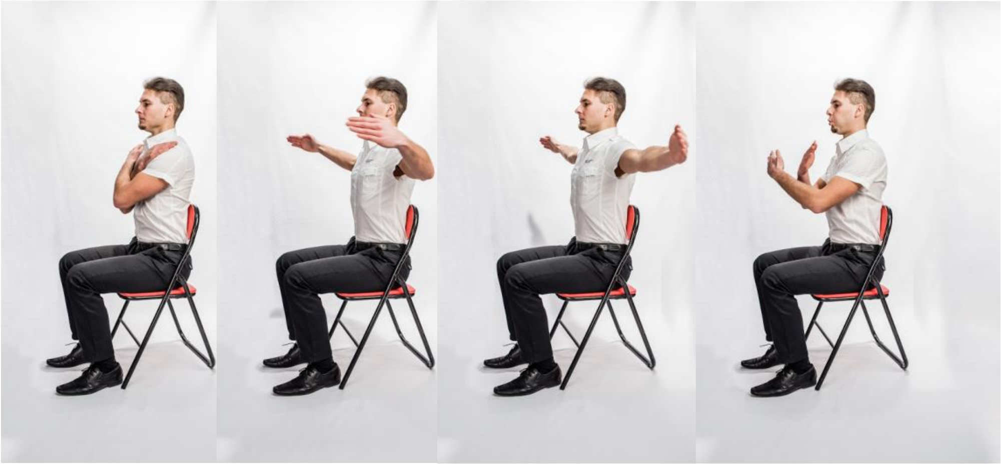

Breathing exercise 3. Both hands should be crossed on the chest. Abduct your arms to the sides while inhaling deeply through your nose, and then, along with a slow, long, and deep exhale through the narrowed mouth, cross your hands over your chest again. Repeat the exercise 6-8 times.

Breathing exercise 4. Place both hands on your abdomen near the navel and press lightly. Take a deep breath through your nose, drawing air "into your belly" so as to use the drawn air to push your hands out against their resistance. The chest should remain motionless. Exhale long and slowly through narrowed lips. Repeat the exercise 6-8 times.

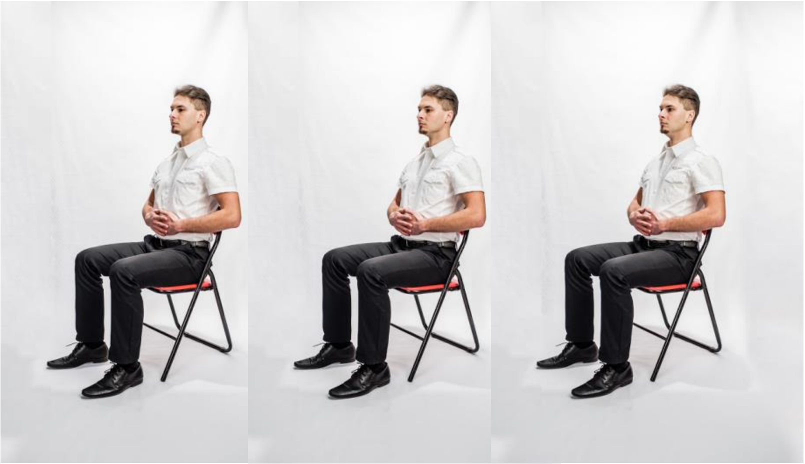

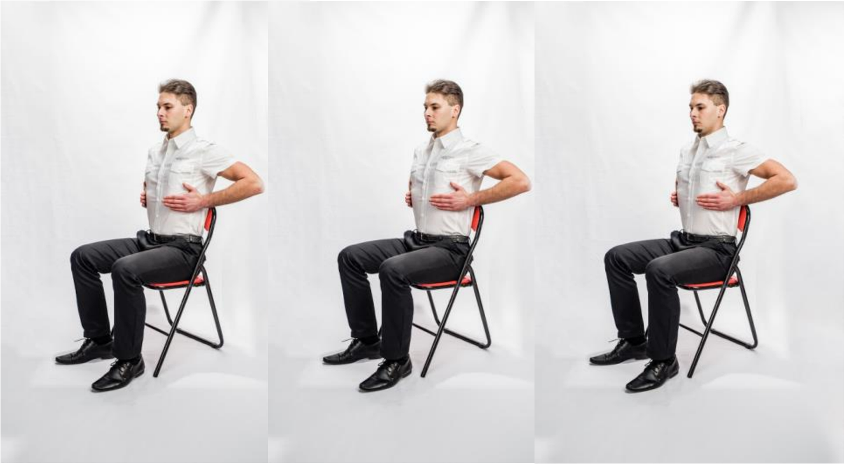

Breathing exercise 5. Place both hands on the side of your chest and press lightly. Elbows should face your sides. Take a deep breath through your nose and use the drawn air to push the lower ribs out against the resistance of your hand. The abdomen should remain flat throughout the entire exercise. Do not pull in or push out your belly. Exhale long and slowly through narrowed lips. Repeat the exercise 6-8 times.

FINAL PART (5 – 15 MINUTES)

Slow down to a gentle, walking pace and calm your breathing down.

Relax your arms and legs by gently shaking them.

1. Extend your upper limb out in front of you straight at the elbow. Then grab the elbow with your other hand and bring it to your chest. Feel the stretch in the back, upper part of your arm. Hold the position for several seconds and breathe freely at the same time. Don't twist your torso. Repeat the exercise 2 – 3 times for both sides of the body.

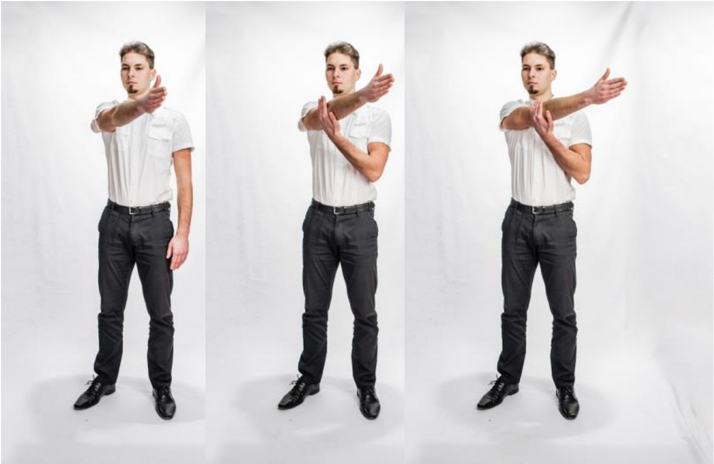

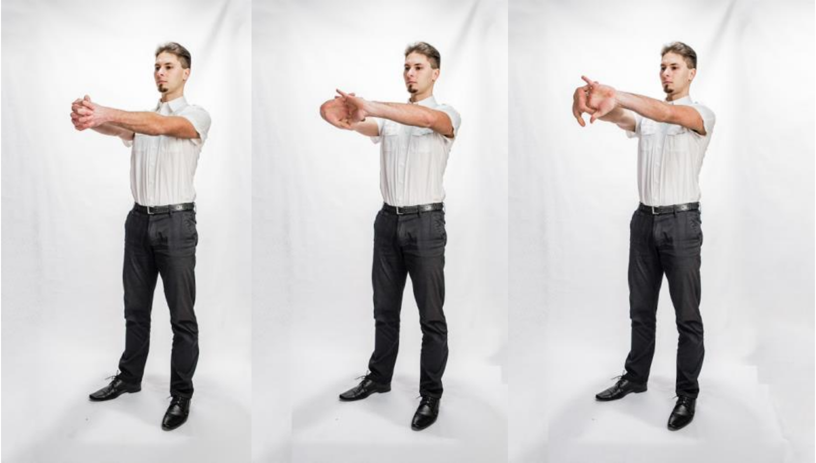

2. Interlock the fingers of both hands together. Turn your hands over and feel the stretch in the forearm area. Hold the position for a few seconds and breathe freely at the same time. Repeat the exercise 2 – 3 times.

Relax your arms and legs by gently shaking them.

3. Extend your right leg straightened at the knee and place it on the heel. If possible, place your heel on a slight elevation (step, stool, curb, etc.). Straighten your back, place both hands on your right knee, and lean forward slightly. Feel the stretch on the back of your thigh. Hold the position for a few seconds and breathe freely at the same time. Repeat the exercise 2 – 3 times for both sides of the body.

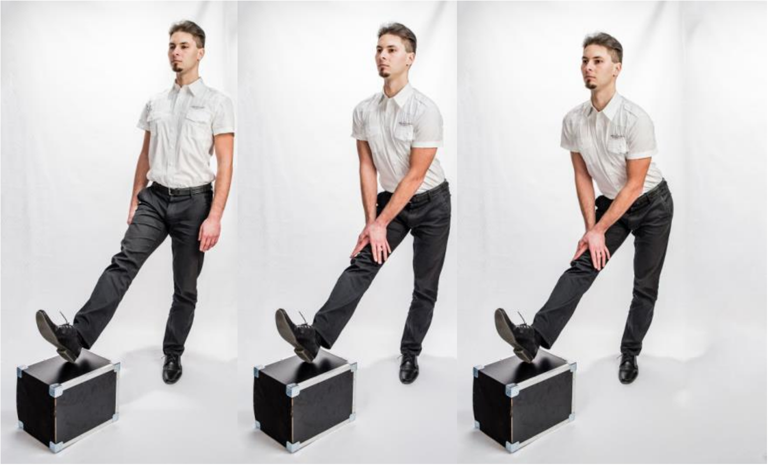

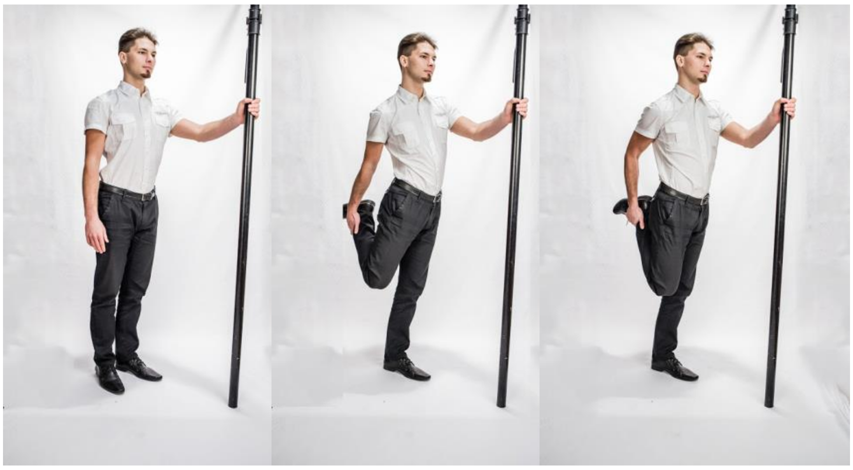

4. Find a place to hold onto something. Bend your right leg at the knee and bring your heel to your buttock with your right hand. Feel the stretch on the front side of your thigh. Do not let the bent knee slide excessively forward. Hold the position for a few seconds and breathe freely at the same time. Repeat the exercise 2 – 3 times for both sides of the body.

5. Lean your front against something solid (wall, door frame, tree, etc.). Pull your right leg back and rest on your toes. Try to put your heel on the ground as well and feel the stretch in your calf. Hold the position for a few seconds and breathe freely at the same time. Repeat the exercise 2 – 3 times for both sides of the body.

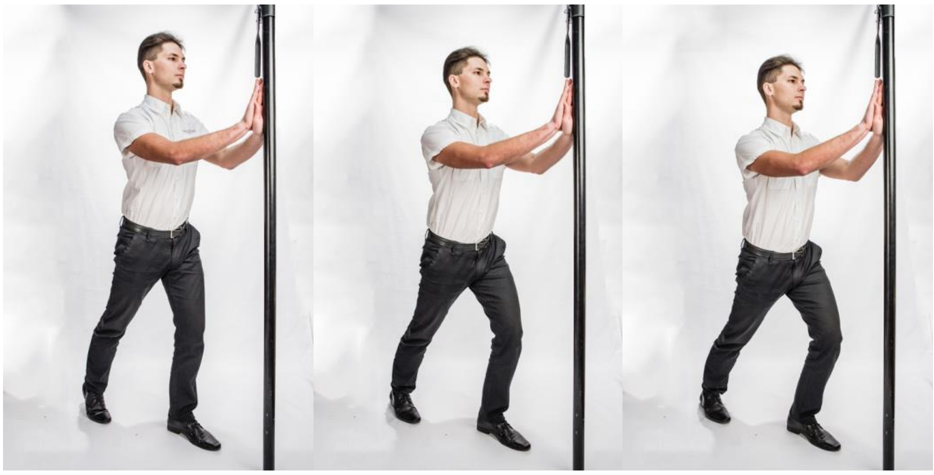

Relax your arms and legs by gently shaking them.

ATTENTION! Before and after training, measure your blood pressure, heart rate, training time, and fatigue level on a ten-point fatigue scale (0-10). If you are using additional activity monitoring devices (accelerometer, smartphone application for monitoring physical activity, pulse oximeter, etc.), gather this data from the devices and record it in the **"Self-control diary"**.
